# Exploring green purchasing intentions and behaviours among Vietnamese Generation Z: A perspective from the theory of planned behaviour

**DOI:** 10.1371/journal.pone.0323879

**Published:** 2025-05-28

**Authors:** Quang-Huy Ngo, Thanh-Dung Nguyen, Nhu-Binh Phan

**Affiliations:** Business Department, FPT Greenwich, FPT University, Can Tho City, Vietnam; International University - Vietnam National University Ho Chi Minh City, VIET NAM

## Abstract

As environmental degradation escalates, the critical need to understand green purchasing intentions and behaviours among Vietnamese Generation Z becomes increasingly urgent. Although the Theory of Planned Behavior (TPB) has been extensively applied to study pro-environmental behaviours, there remain discrepancies in how green attitudes, green subjective norms, and green perceived behavioural control influence green purchasing intentions and green purchasing behaviours. This study aims to clarify these relationships within the unique socio-economic and cultural context of Vietnamese Generation Z, a demographic influenced by collectivistic cultural values, generational characteristics, and dynamic economic conditions. These factors may reshape the conventional dynamics of TPB. Utilising quantitative methodologies, this research analysed responses from 237 Vietnamese Generation Z consumers through structural equation modelling to assess the impacts of green attitude, green subjective norms, and green perceived behavioural control on green purchasing intentions and green purchasing behaviours, particularly focusing on the mediating role of green purchasing intentions. The findings demonstrate that green attitude, green subjective norms, and green perceived behavioural control significantly affect both green purchasing intentions and green purchasing behaviours, thereby confirming the mediating influence of green purchasing intentions. This research reaffirms TPB’s relevance in Vietnam’s distinct cultural and economic environment while contributing to the broader TPB literature by exploring the mediating effects among key variables. These results also underscore the need for policymakers and businesses to create community-oriented environmental programs and tailor marketing strategies to enhance pro-environmental purchasing among young consumers.

## 1. Introduction

The rise in global consumption has fueled environmental destruction and waste generation, both of which are major contributors to this degradation [1]. Personal consumption is widely recognised as one of the most significant threats to the environment [2]. Green purchasing behaviour emphasises the selection of recyclable and eco-friendly products, conserves resources, and supports the health of the natural environment [3]. There is an urgent need for scholars to illuminate the drivers behind individuals’ adoption of such behaviours [4–7]. The Theory of Planned Behaviour (TPB) [8] serves as a valuable theoretical lens to explore the determinants of green purchasing behaviours. In the literature on pro-environmental behaviours, this theory posits that green attitude, green subjective norms, and green perceived behavioural control determine green purchasing intentions, which in turn significantly influence green purchasing behaviours [9,10].

However, the existing literature reveals substantial inconsistencies in the relationships among these key TPB antecedents when predicting green purchasing intentions and green purchasing behaviours [11–19]. Similarly, the inconsistencies extend to the relationships between green attitude, green subjective norms, green perceived behavioural control, and green purchasing intentions [11,12,20–27], as well as the relationship between green purchasing intentions and green purchasing behaviours [12,14,24,28].

These contradictory findings underscore a key limitation in the current TPB literature in predicting green behaviours, particularly when contextual factors such as generational differences, cultural norms, and economic conditions are taken into account. This study addresses these gaps by examining TPB’s applicability among Generation Z consumers in Vietnam, a collectivistic and emerging market where such factors may uniquely shape green consumption. Hence, this study proposes the following research questions.

Q1: Whether green attitude, green subjective norms, and green perceived behavioural control respectively promote green purchasing intentions and behaviours of Vietnamese Generation Z consumers?

Q2: Whether green purchasing intentions promote green purchasing behaviours of Vietnamese Generation Z consumers?

Although TPB does not inherently assume mediation among its core variables [29], recent empirical results [30–32] adequately provide evidence supporting the mediating effects of various key variables within the framework. In alignment with those empirical results, this study expects the mediating effects of green purchasing intentions on the impact of green attitude, green subjective norms, and green perceived behavioural control, respectively, on green purchasing behaviours. Hence, the last research question is as follows.

Q3: Whether green purchasing intentions mediate the impact of green attitude, green subjective norms, and green perceived behavioural control on green purchasing behaviours in Vietnamese Generation Z consumers?

To shed light on those research questions, this study executes a quantitative approach to examine the proposed research model. Based on the theoretical lens of TPB, this study hypothesised that three independent variables, such as green attitude, green subjective norms, and green perceived behavioural control, significantly promote green purchasing intentions, and green purchasing intentions significantly enhance green purchasing behaviours. Furthermore, the hypotheses of green purchasing intentions as mediators are also proposed. The study collected data from 237 Vietnamese Generation Z participants to test this proposed research model. Partial least squared structural equation modelling (PLS-SEM) is the statistical approach used to examine the sample. The findings reveal that the three factors, such as green attitude, green subjective norms, and green perceived behavioural control, directly and significantly promote green purchasing intentions as well as green purchasing behaviours. Besides, the link between green purchasing intentions and green purchasing behaviours was revealed to be significant. Lastly, based on the findings, green purchasing intentions serve as a mediator in the direct impact of these three factors and these behavioural outcomes.

The results significantly contribute to the literature in two folds. Firstly, this study follows the call of YurievDahmen [9] to demonstrates the contextual adaptations of the original TPB in diverse settings. Secondly, this study contributes to the growing body of TPB knowledge on the mediating roles of various key variables in the TPB framework [30–32]. On a practical level, the findings of this study provide actionable insights for both policymakers and firms which target the promotion of green purchasing behaviours among Vietnamese Generation Z.

## 2. Theoretical lens and framework

### 2.1. Theory of planned behaviour

Through expanding the Theory of Reasoned Action, TPB includes the concept of perceived behavioural control as a factor determining behavioural intention, which determines a certain behaviour [8]. TPB distinguishes three primary factors that govern human behaviour: beliefs about behaviour, norms, and control. According to studies [8, 33], these elements significantly affect behavioural intentions and result in certain behaviours. There has been substantial testing of the TPB model and implementation in a variety of domains, which establishes it as a foundational paradigm in behavioural science research [34,35].

In the TPB framework, an attitude refers to the assessment of a person (e.g., favourable or unfavourable) on engaging in a particular conduct, which reflects their inclination toward that particular action. Behavioural beliefs, or views about the expected results of the behaviour, and outcome evaluations, or assessments of the attractiveness of these results, combine to generate this attitude. Research has methodically confirmed the connection between attitude and behavioural intention, which demonstrates the significance of attitude as a key determinant of intention [33,35–37].

The sense of social pressure to participate in or abstain from particular conduct, which is an important component of the TPB, refers to the subjective norm. Normative views, which stand for a person’s assessments of how significant other people think they should act and their incentive to do so, influence this pressure [33, 38]. Numerous empirical studies have consistently demonstrated that subjective norms significantly predict behavioural intention, especially in social contexts where others’ opinions are highly influential [37, 39, 40].

Perceived behavioural control, or the ability to judge the constraint degree in conducting an action, is another element of the TPB’s framework. Perceived power and control beliefs influence this perception [33, 41]. Perceived availability of resources like time, money, or opportunities that could help or hinder the activity is a component of control beliefs. Conversely, perceived power is a person’s conviction in their capacity to overcome these problems [41]. According to studies, perceived behavioural control has a crucial role in predicting intention and action, especially when people are faced with uncontrolled constraints [42–44].

According to this framework, an actual behaviour is significantly determined by behavioural intention. In addition, this intention is significantly affected by attitude, subjective norm, and the perception of behaviour as well as their interaction [42]. When these three factors are sufficient, a person’s tendency to participate in the activities grows, which generally culminates in actual conduct [8, 35]. The association between intention and behaviour has been extensively validated across various domains, including health, environmental, and consumer behaviour [45–47].

### 2.2.  Theory of planned behaviour in green contexts

In the green consumerism literature, green purchasing intentions and green purchasing behaviours are central topics of study because understanding the underlying drivers and barriers of these intentions and behaviours is crucial for developing effective green marketing strategies aimed at promoting environmental sustainability [48,49]. A version of TPB, which is used in green consumerism contexts, was suggested to shed light on the antecedents of these intentions and behaviours. According to this version, three variables, green attitude, green subjective norms, and green perceived behavioural control, significantly determine green purchasing intentions and green purchasing behaviours [9,10].

#### 2.2.1 . Green attitude.

In the realm of consumer behaviour, green attitude refers to an individual’s tendency to prioritise environmentally responsible choices [50]. This attitude is characterised by a propensity to support and engage in sustainable behaviours that contribute to reducing pollution and positively impacting environmental conservation [51]. Essentially, it represents a mindset or belief system that motivates individuals to actively endorse and participate in environmentally friendly practices [52].

#### 2.2.2 . Green subjective norms.

The perceived social pressure, which people feel when participating in green purchasing behaviours, is mentioned in green subjective norms, and it affects the intent to purchase ecologically friendly products [53]. This approach takes into account the influence of social expectations, which are often shaped by the beliefs and values of important people, including family, friends, and the general public, on a person’s decision to participate in activities relating to the sustainability of the environment [54]. Furthermore, green subjective norms reflect how social norms affect a person’s choice to engage in green activities, such as using urban green spaces, where their actions are guided by the expectations and behaviours of those within their social circle [55].

#### 2.2.3 . Green perceived behavioural control.

It encompasses the evaluation of an individual’s capacity to participate in environmentally conscious purchasing behaviours [10]. This perception is shaped by various internal and external elements that can either support or impede such actions [7]. It involves individuals evaluating their ability to carry out eco-friendly practices, taking into account the accessibility of resources and external circumstances [56]. Moreover, this idea highlights how much customers think they have the necessary resources and ability to make purchases that will not negatively impact the environment. This belief incorporates considerations of potential facilitating factors or barriers that may influence their decisions [10].

#### 2.2.4 . Green purchasing intentions.

It refers to the consuming tendency to purchase environmentally friendly products/services. This intention is shaped by a number of variables, which also affect the individual’s actual purchase decisions. These variables include personal attitudes, social conventions, and the perception of the ability to engage in such conduct [10]. This concept also encompasses the consumer’s willingness or probability of buying eco-friendly products. Such intentions are driven by the individual’s knowledge about these products, their attitudes towards them, and their perceptions of their environmental impact [57]. Furthermore, green purchasing intentions reflect a consumer’s propensity to make environmentally conscious buying choices. This propensity is influenced by a person’s environmental beliefs, exposure to environmental media coverage, and assessment of the significance of sustainable activities [58].

#### 2.2.5 . Green purchasing behaviours.

Green purchasing behaviour specifically involves buying eco-friendly products that are recyclable, conserve resources, and are beneficial to the natural environment. Consumer purchasing behaviour describes the process through which individuals select products, services, or ideas to meet specific needs [59]. A subset of this idea called green purchasing behaviours deals with the use of biodegradable or environmentally friendly items as well as those that take the environment’s effects into account [60]. This behaviour includes choosing and obtaining goods and services with the intention of minimising adverse influences on the environment at every stage of their lifespan, including production, transportation, use, recycling, and disposal [61]. It is frequently linked to ethical, sustainable, ecologically friendly, and responsible shopping habits [62]. Green purchasing behaviours manifest in various ways, such as opting for energy-efficient products, avoiding excessively packaged items, choosing biodegradable and recyclable goods, and making efforts to reduce pollution [62].

#### 2.2.6 . The role of contextual factors on TPB in green consumerism.

Contextual factors, such as national culture, generational differences, and economic development, play a significant role in shaping how individuals engage with green consumerism, especially when examined through the lens of TPB. Moreover, within a collectivistic culture, generation as Generation Z, and economic development of emerging economies, there are concerns about using the TPB framework due to the following contrasting arguments.

First, collectivism potentially affects the interrelationship between key variables within the TPB in green consumerism contexts. In collectivist cultures, individuals are more likely to align their actions with the desires of the collective because maintaining social harmony and securing group approval are key priorities [63]. Collectivistic culture significantly increases conformity because collective thinking is a more powerful driver of behaviour than individual values [64]. There are two contrasting arguments relating to the impact of conformity on green intentions and behaviours. On the one hand, individuals living in collectivistic cultures may commit to green behaviours due to the strong emphasis on community welfare and shared social norms, which encourage collective actions to protect the environment for future generations [65]. On the other hand, conformity significantly causes collectivistic individuals to exert non-green behaviours in an extravagant atmosphere [66]. When extravagant consumption is the symbol of social status, conformity significantly drives individuals to exert non-green behaviour to gain social acceptance and avoid social exclusion [67].

Second, generational differences, particularly the characteristics of Generation Z, also play a critical role in the variations in the interrelationship between main variables within TPB in green consumerism contexts. Generation Z individuals show significant environmental consciousness and proactive sustainability behaviours because of their unique combination of early exposure to climate issues and a deep sense of social and intergenerational responsibility, which produces more commitment to environmental sustainability than previous generations [68]. Their green purchasing decisions align with their vich are driven by a strong sense of responsibility for the environment and a commitment to reducing their ecological footprint for the sake of future generations [69]. Societal norms, significantly shaped by social media and peer pressure in this generation, influence Generation Z’s green behaviours by fostering a powerful network of environmental advocates and sustainability-driven content, which motivates them to embrace consumerism decisions that align with environmental values [68]. However, Generation Z individuals are price-sensitive and have limited disposable income [70,71]. Furthermore, they are considered as the generation that values money more than prior generations [72]. These characteristics reduce the green intentions as well as the green behaviours of this generation.

Third, regarding the economic development of emerging countries, there are two conflicting arguments when predicting green intentions and behaviours. On the one hand, the economic conditions in these countries may limit the government’s capability to implement policies improving environmental education and raising public environmental awareness as well as providing the necessary infrastructure to support green consumption [4, 73]. In addition, many individuals in emerging economies view green consumption as a low priority, primarily due to the high costs associated with green consumption [74]. On the other hand, some emerging countries are experiencing significant increases portion of the middle class in the population, and therefore, they have more disposable incomes supporting green consumption [75]. Additionally, due to the high accessibility to education, the rise of the middle class in emerging countries significantly contributes to the expansion of public environmental awareness within this country [76]. Finally, these countries have the potential to leapfrog traditional, resource-intensive technologies to adopt more advanced green technologies, which potentially support the infrastructure for green consumption [77].

### 2.3. Research framework

#### 2.3.1 . The link between green attitude and green purchasing behaviours.

Previous research has shown inconsistent findings regarding the relationship between green attitude and green purchasing behaviour. For instance, ZahanChuanmin [11] research on young Bangladeshi individuals over 20 years old found a significant relationship between green attitudes and green purchasing behaviours. Similarly, Chaudhary and Bisai [12] studies of Indian Millennials found similar results. In contrast, Moser [13] an examination of German individuals indicated a non-significant relationship between green attitude and this behaviour. Carrión Bósquez and Arias-Bolzmann [14] Research on Ecuadorian millennials found similar results. The differing contextual factors, such as cultural norms, generational values, and economic conditions in these countries, may account for these mixed results. Those mixed findings pose a concern about the association between green attitudes and green purchasing behaviours within the context of Vietnamese Generation Z consumers.

**Fig 1:**
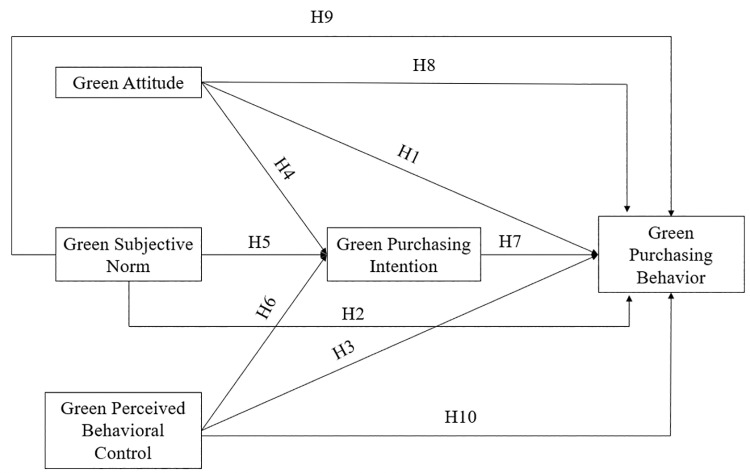
Research model.

Despite these varied findings, this study used TPB to predict a positive impact of green attitudes on green purchasing behaviours among Vietnamese Generation Z consumers (see Fig 1). A green attitude is defined as an individual’s commitment to making environmentally responsible choices [50,51]. Individuals with strong green attitudes are often driven by a moral obligation to select products that support environmental preservation [52]. Moreover, a robust, strong green attitude reinforces an individual’s self-identity as someone dedicated to environmental care, influencing their purchasing decisions to align with their environmental values concept [78,79]. Additionally, individuals with positive, strong green attitudes are more receptive to green marketing efforts, which increases their likelihood of choosing eco-friendly products [80]. Two perspectives potentially explain this significant relationship. The perspective of self-determination suggests that the significant attitude toward green behaviours significantly forms intrinsic motivations, driving individuals to engage in behaviours to reduce the negative impacts on the environment [81,82]. From the views of cognitive dissonance, a significant attitude toward the environment causes individuals to engage in green behaviours to reduce psychological discomfort caused by conflicting attitudes and actions [83]. Based on these theoretical arguments and grounded in TPB, this study proposes the following hypothesis.

**H1**: a positive association between green attitude and green purchasing behaviours exists among Vietnamese Generation Z consumers.

#### 2.3.2 . The link between green subjective norms and green purchasing behaviours.

Research on the relationship between green subjective norms and green purchasing behaviours has yielded inconsistent results. For instance, FieldingMcDonald [15] explored environmental activism and identified a positive link between these variables. BhuttoSoomro [16] also demonstrated a similar positive association when studying young Chinese consumers. Conversely, Moser [13] examined German individuals and reported non-significant effects between these variables. Similarly, Parveen and Ahmad [17] researched public behaviour aimed at reducing urban air pollution in Lahore and reported non-significant findings. One possible explanation for these mixed findings is that the uniqueness of the study contexts, such as cultural norms, generational values, and economic conditions, significantly influences the outcomes. This inconsistency raises questions about the relationship between green subjective norms and green purchasing behaviours in the context of Vietnamese Generation Z consumers.

Despite these varied findings, this study employs TPB to predict a positive impact of green subjective norms and green purchasing behaviours among Vietnamese Generation Z consumers. According to HamJeger [53], green subjective norms generate social pressure to conform to environmentally responsible practices. XuDu [54] suggest that when individuals perceive pressure from their peers or social expectations related to the purchasing of eco-friendly products, they may feel compelled to comply to gain social approval. Additionally, WanShen [55] noted that the desire to avoid negative judgment or criticism from others serves as an additional motivator for individuals to make green purchasing choices. Furthermore, Leonidou and Skarmeas [84] observed that being surrounded by others who prioritise sustainability can reinforce these norms, thereby making green behaviour more habitual and socially reinforced. There are two perspectives explaining this relationship. According to social influence theory, individuals are driven to adopt green behaviours that are commonly accepted within their social groups to achieve social acceptance and evade penalties [85].

Furthermore, from the perspective of social identity, social norms that match individual actions with their group’s values and expectations strengthen green identity and a sense of community, leading to green behaviours [86]. Given these arguments and grounded by the TPB, green subjective norms are expected to positively influence green purchasing behaviours among Vietnamese Generation Z consumers (see Fig 1). Based on this argument, the study posits the subsequent hypothesis.

**H2**: A positive association between green subjective norms and green purchasing behaviours exists among Vietnamese Generation Z consumers.

#### 2.3.3.  The link between green perceived behavioural control and green purchasing behaviours.

Research on the relationship between green perceived behavioural control and green purchasing behaviours has produced mixed findings among scholars. Chen and Chai [18] observed a strong positive relationship between green perceived behavioural control and green purchasing behaviours in a sample of Malaysian undergraduate students. Similarly, BhuttoSoomro [16] demonstrated comparable results in the context of young consumers purchasing environmentally friendly appliances. In contrast, NamDong [19] found no significant association in the context of U.S. individuals purchasing green sportswear. And Chaudhary and Bisai [12] reported similar non-significant findings among Indian Millennials. These inconsistencies suggest that cultural norms, generational values, and economic conditions in different study contexts may influence these outcomes. This variability raises questions about the impact of green perceived behavioural control on green purchasing behaviours, specifically among Vietnamese Generation Z consumers.

TPB provides a robust theoretical framework to support a positive link between green perceived behavioural control and green purchasing behaviours. Green perceived behavioural control instils confidence in individuals and, therefore, empowers individuals to exert environmentally friendly choices [7, 56]. Customers potentially execute their intentions to purchase environmentally oriented products due to their sense of control over the necessary resources, information, and opportunities [10]. A high level of control over purchasing environmentally friendly products enables individuals to engage in green purchasing behaviours with greater ease and feasibility [84]. Furthermore, this strong sense of control also leads to greater consistency in green purchasing because individuals are better equipped to overcome barriers associated with green behaviours [87]. Based on these arguments and supported by the TPB, green perceived behavioural control is expected to enhance green purchasing behaviours in the contexts of Vietnamese Generation Z consumers (see Fig 1). Accordingly, the study posits the subsequent hypothesis.

**H3**: A positive association between green perceived behavioural control and green purchasing behaviours exists among Vietnamese Generation Z consumers.

#### 2.3.4 . The link between green attitude and green purchasing intentions.

The investigation into the correlation between green attitudes and green purchasing intentions has shown mixed outcomes across different studies. SynodinosMoraes [20] explored this relationship among South Korean Generation Y consumers with regard to food products and confirmed a strong positive association. Similarly, ZahanChuanmin [11] research on young Bangladeshi individuals over 20 years old found a significant relationship between green attitudes and green purchasing intentions. On the other hand, Sharma and Foropon [21] examined the Indian individuals’ green purchasing intentions and found no significant impact. Similarly, AsifZhongfu [22] investigated Pakistani individuals’s green consumption was investigated and revealed no significant effects. These divergent findings suggest that variables such as cultural norms, generational values, and economic conditions may significantly shape these relationships. This leads to further examination of whether green attitude influences green purchasing intentions among Vietnamese Generation Z consumers.

Utilising TPB as a theoretical backdrop, a strong positive link is anticipated between green attitude and green purchasing intentions. Specifically, individuals who hold favourable views towards environmental conservation are typically more predisposed to engage in behaviours aligned with these values, such as purchasing sustainable products, as highlighted by recent research [50, 88]. [89] also noted that a pronounced environmental attitude catalyses the formation of corresponding purchase intentions. This phenomenon can be attributed to two primary reasons. First, individuals who cherish environmental values tend to develop firm attitudes that catalyse strong intentions [90]. Second, a profound emotional engagement with environmental issues often propels a positive attitude, which in turn fosters strong purchase intentions [91]. Therefore, the intensity of green attitude is expected to have a notable influence on green purchasing intentions in the context of Vietnamese Generation Z consumers (see Fig 1). Consequently, the study posits the subsequent hypothesis.

**H4**: A positive association between green attitude and green purchasing intentions exists among Vietnamese Generation Z consumers.

#### 2.3.5 . The link between green subjective norms and green purchasing intentions.

The findings of research on the connection between green subjective norms and green purchasing intentions have been mixed. BhuttoZeng [23] researched on young Chinese consumers and found the significant and positive relationship between green subjective norms and green purchasing intentions. MaichumParichatnon [24] examined Thai consumers’ propensity to buy green products and observed the same effects. However, Chaudhary and Bisai [12] studies on Indian Millennials found no significant effect. Also, ZahanChuanmin [11] research on Bangladeshi young individuals over 20 years old found no significant relationship between these variables. These varying results indicate that factors like cultural standards, values across different generations, and economic situations might greatly influence these connections. This prompts additional investigation into whether green subjective norms affect green purchasing intentions among Generation Z consumers in Vietnam.

TPB offers a strong theoretical basis to affirm a positive relationship between green subjective norms and green purchasing intentions. Individuals are under pressure to intend to purchase environmentally friendly products when their family, friends, or society expects them to behave in a manner that respects the environment [54, 92]. This social pressure, referred to as green subjective norms, motivates individuals to align their intention to exert purchasing decisions with these expectations [53, 93]. From the perspective of social influence, the perception of behaviours that are prevalent and accepted within social groups motivates individuals to adopt these behaviours in order to gain social acceptance and avoid sanction [85]. Besides, on the view of social identity, social norms, which align individual behaviours with the values and expectations of their social group, reinforce green identity and a sense of belonging, thereby influencing green purchasing intentions [86]. Given these arguments, a positive and significant relationship between green subjective norms and green purchasing intentions is expected among Vietnamese Generation Z consumers (see Fig 1). Hence, the study proposes the following hypothesis.

**H5**: A positive association between green subjective norms and green purchasing intentions exists among Vietnamese Generation Z consumers.

#### 2.3.6. The link between green perceived behavioural control and green purchasing intentions.

Mixed findings have been found in the research on the link between green perceived behavioural control and green purchasing intentions. For instance, Yoon and Joung [25] investigated South Korean individuals’ green purchasing intentions and showed a positive and significant relationship. Also, WangZhang [26] examined Chinese millennial individuals’ car purchasing and found the same result. However, JainGupta [27] examined the green consumption of Indian Millennials and found no significant relationship between green perceived behavioural control and green purchasing intentions. Similarly, Chaudhary and Bisai [12] investigated the Indian Millennials and found no evidence supporting this relationship. These inconsistent outcomes suggest that elements such as cultural norms, generational values, and economic conditions can significantly impact these relationships. This leads to further research into whether green perceived behavioural control influences green purchasing intentions among Generation Z consumers in Vietnam.

A solid theoretical foundation that supports a positive correlation between green perceived behavioural control and green purchasing intentions is established by TPB. In particular, green perceived behavioural control consults to the belief in a person’s ability to make environmentally responsible choices based on their resources, knowledge, and opportunities. This perception of control enhances their confidence in purchasing eco-friendly products [94,95]. Sun and Wang [96] argued that this perceived control strengthens the intention to purchase products having sustainable characteristics. From the perspective of self-efficacy, the perception of behavioural control enhances individuals’ confidence in their ability to successfully choose and use eco-friendly products, thereby increasing their intention to purchase green products [54].

Furthermore, the perspective of locus of control argues that individuals possess a strong internal locus of control relating to the environment, showing their positive intention toward the purchase of green products because of the perception of their actions contributing to environmental well-being [97]. Consequently, it is expected to observe the positive and significant link between green perceived behavioural control and green purchasing intentions (see Fig 1). Hence, the following hypothesis describes this expectation.

**H6**: A positive association between green perceived behavioural control and green purchasing intentions exists among Vietnamese Generation Z consumers.

#### 2.3.7 . The link between green purchasing intentions and green purchasing behaviours.

The results of studies on the link between green purchasing intentions and green purchasing behaviours have been inconsistent. Chaudhary and Bisai [12] observed significant and positive effects of green purchasing intentions on the behaviours among Indian Millennials. MaichumParichatnon [24] examined the Thai individual’s green consumption and found significant effects between these two variables. However, Carrión Bósquez and Arias-Bolzmann [14] indicated a non-significant relationship among Ecuadorian millennials. Echegaray and Hansstein [28] also demonstrated the same effects among Brazilian individuals. The mixed results may be attributed to varying contextual factors like cultural norms, generational values, and economic conditions in these countries. These mixed findings raise questions about the relationship between green purchasing intentions and green purchasing behaviours among Vietnamese Generation Z consumers.

Despite the diverse results observed in previous studies, this investigation applies TPB to forecast a positive influence of green purchasing intentions, which significantly impact green purchasing behaviours among Vietnamese Generation Z consumers. Specifically, green purchasing intentions are defined as a deliberate and conscious decision to prioritise environmentally friendly products, which reflects an active commitment that significantly influences green behaviour [10, 57, 98]. Individuals who exhibit strong green purchasing intentions are likely to translate these intentions into actual purchasing decisions because these intentions serve as a decisive guiding force in their consumer behaviour [58]. This intentionality is considered a deep-seated commitment to environmental sustainability, which consistently drives individuals to engage in green behaviours [99]. Moreover, green purchasing intentions are intrinsically linked to a sense of moral obligation, which has been demonstrated to significantly promote green consumption behaviours [65, 100]. This moral obligation encourages individuals to integrate ethical considerations and social responsibility into their purchasing decisions, which inclines them to purchase eco-friendly products [97]. Therefore, it can be posited that green purchasing intentions exert a positive effect on green purchasing behaviours within the context of Vietnamese Generation Z consumers (see Fig 1). Based on these theoretical and empirical insights, the study proposes the following hypothesis.

**H7**: A positive association between green purchasing intentions and green purchasing behaviours exists among Vietnamese Generation Z consumers.

#### 2.3.8 . The mediating role of green purchasing intentions.

Prior research reveals inconsistent findings regarding the impact of green attitude, green subjective norms, and green perceived behavioural control on green purchasing behaviours. In addition to the influential role of cultural and generational values and economic development, an underlying mechanism appears to mediate these impacts. Particularly, according to TPB, green purchasing intentions may serve as a mediator in these relationships [41]. First, a green attitude, which reflects an individual’s commitment to making environmentally responsible choices, sufficiently translates into a strong intention toward the environment [10, 50]. Individuals with positive environmental attitudes are likely to develop strong intentions to engage in green purchasing [101]. These intentions act as a bridge, converting attitudes into actual green purchasing behaviours [12, 102]. Second, green subjective norms represent perceived social pressures to engage in green behaviours [53]. When individuals perceive expectations from significant others to purchase green products, they are likely to form intentions sufficient to comply with these expectations [54]. These intentions play a significant role in materialising into actual green purchasing behaviours [15,16]. Third, green perceived behavioural control refers to an individual’s perception of their ability to perform environmentally friendly behaviours [10]. High perceived control boosts confidence in one’s ability to purchase green products, therefore leading to strong green purchasing intentions, which facilitates the actual purchasing of green products (Yoon & Joung, 2019)[10]. Prior studies suggest that perceived behavioural control influences intentions, which in turn drive green purchasing behaviours (Bhutto et al., 2022; Chen & Chai, 2010). Therefore, green purchasing intentions are posited as a crucial variable that bridges the gap between green attitude, green subjective norms, green perceived behavioural control, and green purchasing behaviours (see Fig 1). Building on these arguments, the study expects to validate the following hypotheses.

**H8**: The association between green attitude and green purchasing behaviours is mediated by green purchasing intentions among Vietnamese Generation Z consumers.

**H9**: The association between green subjective norms and green purchasing behaviours is mediated by green purchasing intentions among Vietnamese Generation Z consumers.

**H10**: The association between green perceived behavioural control and green purchasing behaviours is mediated by green purchasing intentions among Vietnamese Generation Z consumers.

## 3. Methods

### 3.1. Data collection

This research investigated green consumption behaviour among Generation Z in Vietnam. The study employs a convenience sampling approach by using Facebook as the primary data collection platform for several compelling reasons. First, the target demographic, classified as Generation Z, is noted for high internet literacy and knowledgeability [103, 104]. In Vietnam, Facebook enjoys widespread popularity among this group, with approximately 95% actively using the platform [105], which minimises concerns of selection bias. According to Schneider and Harknett [106], using Facebook for data collection provides significant advantages. Facebook’s targeted advertisement capabilities allow for precise sample targeting and rapid data gathering, which is particularly beneficial for research opportunities like this one. The low cost of this approach, combined with the ability to quickly adjust sampling parameters in response to real-time data, enhances the efficiency and adaptability of the research methodology. These strengths make Facebook a valuable tool for efficiently reaching and studying the Vietnamese Generation Z population in Vietnam. Ethical approval for this study was granted by the Head of the Business Department at FPT-Greenwich, FPT University, Can Tho campus (Approval No. FPTGREENWICH/Ethic/2024.03.01).

To identify relevant participants, the study targeted specific Facebook groups using keywords such as #TieuDungXanh (Green Consumption), #TieuDungBenVung (Sustainable Consumption), #SongXanh (Green Living), #BaoVeMoiTruong (Protect the Environment), and #HanhDongViMoiTruong (Act for the Environment). A Google Forms-powered online survey was extended to respondents with the invitation to participate. The survey, exclusively targeting Vietnamese Generation Z, was accompanied by an informed written consent form detailing the study’s objectives, methodology, potential risks and benefits, confidentiality measures, and participant rights. Participants were promised full confidentiality and anonymity, and they could withdraw at any moment with no obligation. No incentives were offered for participation. Singer and Bossarte [107] argued that incentives, while effective in increasing participation rates, may raise ethical concerns if they create undue influence, therefore potentially compromising the voluntary nature of consent.

Following the ethical concerns outlined by HokkeHackworth [108], our study adopts a structured parental consent process to address key issues such as privacy, informed consent, and the protection of minors. The process includes a consent form that explicitly explains the purpose, methods, potential risks, and participant rights in clear and accessible language. This approach ensures that parents or guardians fully comprehend the study’s objectives and the implications of their child’s involvement before providing approval. Besides, this process is also in alignment with the Vietnamese regulatory framework. First, the process adheres to the Vietnamese Children’s Law [109], which mandates parental consent for minors’ involvement in activities affecting their rights and welfare. Second, the process also complies with the Vietnamese Cybersecurity Law [110], which governs online data collection. Hence, this process provides a transparent and reliable framework for safeguarding minors in internet-based research.

Following the recommendations of DurmazDursun [111], this study implemented two approaches to mitigate potential social desirability bias. First, throughout the data collection process, anonymity and confidentiality were rigorously maintained, as respondents are more likely to provide honest answers when assured of privacy. Second, all questions were carefully worded in a neutral and unbiased manner to minimise the likelihood of participants responding in a socially desirable manner.

Besides, to address potential non-response bias, we conducted a t-test comparing early and late responses, and the results indicate the absence of this bias [112]. Additionally, the bias resulting from common method variance was also examined. Harman’s single-factor test indicates the total variance explained by a factor is less than the cut-off value of 0.5. Besides, the examinations of VIFs of inner models were also executed. The results suggest that those VIFs are all less than the threshold of 0.3, meeting the requirement of the absence of this bias [113].

This study employs a cross-sectional survey to gather data on all variables in the model within a single phase. There is typically a concern about the intention-behaviour gap, where intentions may change if there is a significant time lapse between stating intentions and committing to behaviours. However, this concern is mitigated in the context of green consumption. Research suggests that green consumption is often impulsive and characterised by immediate decision-making [114], which lessens the impact of any time-related changes in intentions. Unlike health-related behaviours, dietary habits, and exercise routines, where a prolonged period between intention and action is common [115–117], the immediacy of green consumption decisions contributes to the stability and consistency of this behaviour over time.

This argument aligns with prior research employing cross-sectional surveys in green purchasing contexts, which consistently demonstrate a significant relationship between intention and behaviour [10–12,118–121]. Moreover, several studies using this methodology report substantial explanatory power for behaviours relating to green consumption. For instance, [122] found that the intention alone explained 52.5% of the variance in the behaviour. When subjective norms were included alongside intention, [123]it was reported that these two variables accounted for 58.1% of the total variance in behaviour. Similarly, Duong [124] indicated that combining attitude with intention increased the explained variance in G_PB to 62.23%. Given this evidence, the use of a cross-sectional survey capturing both intention and behaviour at a single time point does not significantly undermine the reliability of the results in this study.

The participant recruitment for this study was conducted between April 1st and May 31st, 2024. After removing incomplete responses and those outside the target age group, 237 valid cases were obtained. This sample size is considered sufficient based on three key perspectives. First, the 10-times rule [125] requires a sample size of at least ten times the maximum number of arrows pointing to any latent variable in the model. Based on this rule, a minimum of 60 cases is required, which is well exceeded in this study. Second, the gamma-exponential method using Monte Carlo simulations [126] suggests a minimum of 146 observations when the path coefficient is unknown. Third, the power table [127] recommends approximately 155 cases for detecting path coefficients between 0.11 and 0.20 at a 5% significance level with 80% power. The selection of 0.11 to 0.20 as the expected range of path coefficients is grounded in prior research on behavioural and social sciences, where small to medium effect sizes are commonly observed [128].

SmartPLS is a widely used software for PLS-SEM analysis because of the ability to analyse complex relationships between variables, especially in fields like social sciences, business, and behavioural studies [129]. It is particularly beneficial when data does not meet strict normality assumptions or when working with small sample sizes [130]. With its user-friendly interface and robust statistical tools, SmartPLS enables researchers to assess measurement models, test hypotheses, and analyse indirect effects efficiently [131]. The latest version, SmartPLS 4, introduces several enhancements, including improved data import capabilities, a redesigned graphical user interface, and advanced reporting features, thereby streamlining the modelling process [132]. Therefore, this study employs SmartPLS 4 for data analysis.

Table 1 provides a detailed demographic profile of the 237 Vietnamese Generation Z participants in this study. The sample shows a significant gender imbalance, with females representing a substantial majority at 76.79% of respondents, while males account for only 23.21%. The age distribution of participants reveals that the majority fall within the younger segments of Generation Z. The largest group, comprising 41.77% of the sample, is aged 15–18 years. This is closely followed by the 18–22 age group, representing 35.86% of participants. Younger teens aged 12–15 make up 16.46% of the sample, while the oldest group, aged 22–27, accounts for just 5.91% of respondents. In terms of educational background, the sample is predominantly composed of students at various levels. High school students form the largest group at 41.77%, closely followed by undergraduates at 39.24%. Secondary school students represent 16.46% of the sample, mirroring the proportion of 12- to 15-year-olds. Only a small fraction, 2.53%, have attained graduate-level education. Regarding marital status, the vast majority of participants (91.98%) are single, which aligns with the young age profile of the sample. A small portion, 8.02%, report being married.

**Table 1:** Respondent’s profile.

Characteristics		Frequencies	Percentage
Gender			
	Male	55	23.21
	Female	182	76.79
Age			
	from 12 to less than15 years old	39	16.46
	from 15 to less than 18 years old	99	41.77
	from 18 to less than 22 years old	85	35.86
	from 22 to less than 27 years old	14	5.91
Education			
	Secondary school	39	16.46
	High school	99	41.77
	Undergraduate	93	39.24
	Graduate	6	2.53
Marital status			
	Single	218	91.98
	Married	19	8.02

### 3.2. Measures

The study employed various measures to assess different aspects of green consumer behaviour. Green attitude (G_ATT) was evaluated using a four-item scale derived from Al-Swidi and Saleh [133] work. For green subjective norms (G_SN), we utilised a four-item measure based on Onel [134]. Drawing upon Chaudhary and Bisai [12], a four-item scale was adopted to gauge green perceived behavioural control (G_PBC). Green purchasing intention (G_PI) was examined using a four-item scale adopted from LaheriLim [135], while green purchasing (G_PB) was assessed using a three-item measure from Joshi and Rahman [136]. These instruments were chosen because they have been intensively tested in various cultural and socio-economic contexts, thereby ensuring their reliability.

Building on prior scholars [7, 137], we utilised demographic parameters such as income (INCOME), education level (EDUCATION), gender (GENDER) and age (AGE), as control variables to underscore their critical role in influencing variations in pro-environmental behaviours. This approach is vital because demographic differences, particularly in age, gender, and education, have been shown to significantly impact the degree of pro-environmental engagement. This controlling approach is sufficient since there is a skew in terms of gender distribution in the sample [138]. They all ensure a robust framework for our analysis.

## 4. Results

### 4.1. Measurement models

Table 2 shows that the measurement models are highly reliable, valid, and fit. The factor loadings for each scale all exceed the acceptable 0.7 [139]. Besides, the values of composite reliability (CR) and Cronbach’s alpha all exceed the 07 cutoff value, which suggests the establishment of reliability [139]. Furthermore, the average variance extracted (AVE) values for all components surpassed 0.5, indicating a sufficient degree of convergent validity [139]. The discriminant validity of the research components is assessed through the heterotrait-monotrait correlation ratio (HTMT).

**Table 2:** Measurement model’s validity and reliability.

Model construct	Measurement Item	Loading	Cronbach Alpha	CR	AVE
G_ATT			0.872	0.912	0.722
	G_ATT_1	0.871			
	G_ATT_2	0.836			
	G_ATT_3	0.845			
	G_ATT_4	0.845			
G_SN			0.866	0.908	0.712
	G_SN_1	0.849			
	G_SN_2	0.832			
	G_SN_3	0.845			
	G_SN_4	0.849			
G_PBC			0.869	0.910	0.717
	G_PBC_1	0.833			
	G_PBC_2	0.850			
	G_PBC_3	0.873			
	G_PBC_4	0.831			
G_PI			0.867	0.909	0.714
	G_PI_1	0.841			
	G_PI_2	0.849			
	G_PI_3	0.858			
	G_PI_4	0.833			
G_PB			0.880	0.917	0.735
	G_PB_1	0.854			
	G_PB_2	0.847			
	G_PB_3	0.872			
	G_PB_4	0.857			

According to Table 3, all HTMT values are less than the strict threshold of 0.85, demonstrating appropriate discriminant validity between the components [140]. These results indicate a clear distinction between the different concepts measured in the study. The overall fit of the measurement models is assessed using two key indices. The Standardized Root Mean Square Residual (SRMR) value is 0.048, which is well within the permitted range of 0.08.

**Table 3:** Measurement model’s discriminant validity.

	G_ATT	G_PB	G_PBC	G_PI	G_SN
G_ATT					
G_PB	0.443				
G_PBC	0.117	0.414			
G_PI	0.330	0.763	0.318		
G_SN	0.088	0.448	0.094	0.345	

Additionally, the Normed Fit Index (NFI) value is 0.873, approaching 1. Both these indices suggest a good fit for the measurement models [127]. In summary, these findings provide strong evidence that the measurement models possess robust reliability, validity, and an appropriate overall fit.

### 4.2. Structural models

The study employed a robust statistical approach to validate its findings, utilising 5,000 bootstrap resamples and generating 95% confidence intervals. Table 4 presents the results, which support all proposed hypotheses (H1 to H10) based on path coefficients and statistical significance.

**Table 4:** Path coefficients and hypothesis testing.

Hypotheses	Relationship	Path coefficient	t-value	p-value	Decisions
H1	G_ATT - > G_PB	0.206	4.368	0.000**	Accepted
H2	G_SN - > G_PB	0.220	4.073	0.000**	Accepted
H3	G_PBC - > G_PB	0.204	4.106	0.000**	Accepted
H4	G_ATT - > G_PI	0.247	3.858	0.000**	Accepted
H5	G_SN - > G_PI	0.271	4.411	0.000**	Accepted
H6	G_PBC - > G_PI	0.239	3.707	0.000**	Accepted
H7	G_PI - > G_PB	0.489	7.544	0.000**	Accepted
H8	G_ATT - > G_PI - > G_PB	0.121	3.184	0.001**	Accepted
H9	G_SN - > G_PI - > G_PB	0.133	3.446	0.001**	Accepted
H10	G_PBC - > G_PI - > G_PB	0.117	3.141	0.002**	Accepted
Controlled	AGE - > G_PB	0.300	2.252	0.024*	Significant
Controlled	EDU - > G_PB	-0.213	1.814	0.070	Not significant
Controlled	GENDER - > G_PB	-0.010	0.095	0.924	Not significant
Controlled	M_S - > G_PB	0.028	0.099	0.921	Not significant

**Note.** *: p < .05, **: p < .01

Hypothesis H1 suggests a significant influence of G_ATT on G_PB, which is confirmed by the results (β = 0.206, p < 0.001). Hypothesis H2 indicates that G_SN significantly affects G_PB, which is demonstrated by the results (β = 0.220, p < 0.001). Hypothesis H3 shows that G_PBC has a significant impact on G_PB, which is supported by the results (β = 0.204, p < 0.001). Hypothesis H4 reveals a significant effect of G_ATT on G_PI, which is supported by a Beta value (β = 0.247, p < 0.001). Hypothesis H5 asserts that G_SN significantly influences G_PI, which is evidenced by the results (β = 0.271, p < 0.001). Hypothesis H6 confirms that G_PBC significantly impacts G_PI, which is indicated by the results (β = 0.239, p < 0.001). Hypothesis H7 demonstrates that G_PI strongly predicts G_PB, which is indicated by the results (β = 0.489, p < 0.001). Additionally, the mediation hypotheses H8, H9, and H10 validate the indirect effects of G_ATT, G_SN, and G_PBC on G_PB through G_PI, which are confirmed by the significant following results (β = 0.121, p < 0.001; β = 0.133, p < 0.001; β = 0.117, p < 0.001), respectively.

## 5. Discussion

First, the study reveals a positive direct effect of green attitude on green purchasing behaviours in the contexts of Vietnamese Generation Z consumers. This finding aligns with some previous research [11,12] but contrasts with others [13,14]. The results demonstrate that collectivistic cultural values, the characteristics of Generation Z, and economic growth in emerging countries significantly influence Generation Z’s eco-friendly attitudes. These attitudes, in turn, impact their green purchasing decisions. In societies with strong collectivistic norms, which prioritise group goals over individual desires, Generation Z consumers are likely to develop stronger eco-conscious attitudes because this generational cohort is known for high environmental awareness [63, 68]. Besides, when emerging markets continue to grow dramatically, the availability and affordability of green products improve [141]. The availability of green products permits consumers to sufficiently translate their eco-friendly attitudes into actual purchasing behaviours [89]. This amalgamation of cultural, generational, and economic effects offers a thorough comprehension of the determinants that propel green consumption among Vietnamese Generation Z consumers.

Second, green subjective norms were indicated to positively and directly affect green purchasing behaviours. It is consistent with some studies [15,16] but contrasting with others [13, 17]. The findings suggest that collectivistic cultural values, the distinctive traits of Generation Z, and the economic development in emerging countries significantly mould Generation Z’s green social norms. These norms, in turn, influence their green purchasing decisions. In collectivistic cultures that value communal goals and collective well-being, such norms strongly resonate with Generation Z’s inherent values, which include high environmental awareness and community-oriented decision-making toward environmental harmonisation [65, 69]. Additionally, the rapid economic development in these countries increases both the visibility and accessibility of green products, further encouraging consumers to make environmentally conscious purchasing choices [89, 141]. This blend of factors collectively strengthens the role of green social norms in guiding purchasing behaviours, underscoring how cultural, generational, and economic elements converge to facilitate sustainable consumption patterns among young consumers.

Third, the results show a positive and direct effect of green perceived behavioural control on green purchasing behaviours. Some researchers support this finding [16, 18] but contradicts others [12, 19]. The study indicates that collectivistic cultural values, the characteristics of Generation Z, and economic advancement in emerging nations significantly shape Generation Z’s green perceived behavioural controls. These controls, in turn, influence their decisions related to green purchasing. In collectivistic cultures, where the group’s needs are often placed above individual desires, Generation Z consumers feel a communal responsibility to make environmentally friendly choices [65, 142]. This sense of responsibility, combined with Generation Z’s natural inclination towards the environment, enhances their perceived ability to effect change through their purchasing decisions [68,69]. Furthermore, because emerging markets develop economically, the increased availability and affordability of green products further empower these consumers to easily engage in green purchasing [89, 141]. This confluence of cultural alignment, generational identity, and economic conditions effectively boosts the perceived behavioural control over sustainable consumer behaviours among Vietnamese Generation Z consumers.

Fourth, the study found positive and direct effects of green attitude on green purchasing intentions. This finding aligns with some previous research [11, 20] but contrasts with others [21,22]. The results suggest that the values of collectivistic culture, the characteristics of Generation Z, and the economic development of emerging countries significantly impact Generation Z’s attitudes, which in turn enhance their intentions for environmentally friendly purchasing. In collectivistic cultures that emphasise the welfare of the community, Generation Z individuals are prone to develop strong pro-environmental attitudes as these align with their societal norms and expectations [65, 69]. These attitudes are further reinforced by the economic growth in emerging countries, which not only increases environmental awareness but also provides greater access to green products [141, 143]. As a result, the synergy between cultural values, generational traits, and economic opportunities creates a robust environment that significantly boosts the green purchasing intentions of young consumers in emerging markets.

Fifth, the study found positive and direct effects of green subjective norms on green purchasing intentions. Some previous research supports this result [23,24] but contrasts with others [11,12]. The findings suggest that the collectivistic cultural values, the distinctive traits of Generation Z, and the economic progression in emerging nations significantly influence Generation Z’s subjective norms, subsequently enhancing their intentions to make environmentally friendly purchases. In cultures where community and collective goals are prioritised, Generation Z’s alignment with these social norms strongly motivates them to adopt green behaviours because this generation significantly has high environmental awareness and is under the pressure of their peers to exert green behaviours [63, 68]. Furthermore, the ongoing economic development in these countries not only increases awareness of environmental issues but also makes green purchasing options more accessible [89, 141, 143]. Thereby, it potentially facilitates the translation of positive social norms relating to environments into concrete intentions to purchase green products. This interplay of cultural, generational, and economic factors effectively cultivates a supportive environment for fostering strong environmental intentions among young consumers in emerging markets.

Sixth, the study found positive and direct effects of green perceived behavioural control on green purchasing intentions. The finding is consistent with some previous research [25,26] while it contrast with other [12, 27]. The results demonstrate that the collectivistic cultural values, the characteristics of Generation Z, and the economic development in emerging countries are crucial factors that influence Generation Z’s perceived behavioural control, subsequently boosting their intentions to purchase environmentally friendly products. In environments where collective values are emphasised, Generation Z consumers feel a stronger sense of efficacy and responsibility towards green behaviours because of the significant alignment of generational values and cultural values toward environmental harmonisation [65, 68, 69]. Additionally, significant economic growth in emerging countries provides the necessary resources and access to green products [141]. This synergy among cultural, generational, and economic factors significantly strengthens Vietnamese Generation Z consumers’ ability to act on their intentions toward purchasing green products.

Seven, the study demonstrated a positive and direct effect of green purchasing intentions on green purchasing behaviours. This finding aligns with some previous research [12, 24] but contrasts with other studies [14, 28]. The results reveal that the collectivistic cultural values, the characteristics of Generation Z, and the economic development in emerging countries are crucial factors that shape Generation Z’s intentions toward green purchasing, which in turn impacts their actual decision to make green purchases. In collectivistic cultures, where communal goals and collective benefits are highly valued, Generation Z consumers may develop strong intentions to engage in environmentally friendly purchasing [143]. These intentions are significantly supported by Generation Z’s innate environmental awareness and their tendency to prioritise environmental harmonisation [68,69]. Furthermore, as emerging markets grow economically, they provide more opportunities (e.g., increasing disposable incomes and environmental awareness) and green resources that enable and encourage consumers to act on their green intentions to materialise green behaviours [75, 76, 89, 141]. This interplay between cultural, generational, and economic influences ensures that intentions translate into actual purchasing behaviour, reinforcing the pathway from intentions to actions in the context of Vietnamese Generation Z consumers.

Lastly, this study demonstrates the mediating effects of green purchasing intentions on the relationship between green attitude, green subjective norms, green perceived behavioural control respectively and green purchasing behaviours. This finding implies that in the context of Vietnamese Generation Z individuals, the intention to purchase green products plays a critical role in translating attitudes, social norms, and perceived behavioural controls toward the environment into actual purchasing behaviours relating the green products. This may be due to the collectivistic cultural values, which prioritise community and long-term sustainability, aligning with the inherent characteristics of Generation Z who are socially aware and proactive about environmental issues [65, 68, 69]. Additionally, the economic development in emerging countries like Vietnam is linked to increasing environmental awareness. It provides the necessary infrastructure and market conditions that allow these intentions to be effectively converted into green purchasing actions [75, 76, 89, 141]. This integration of intentions as a mediator underscores the complex yet definitive path through which internal and external influences converge to drive green purchasing among Vietnamese Generation Z consumers.

### 5.1. Theoretical contributions

The results significantly contribute to the literature in two folds. First, Ajzen [144] recently argued that the original TPB framework adequately predicts intention and behaviours due to the assumption of sufficiency. Therefore, scholars do not necessarily add more variables to the framework. Besides, adding new variables to the original framework raises significant concerns due to the risk of over-fitting and the conceptual overlap between additional variables and TPB variables, thereby reducing the relevance of TPB [144, 145]. In the literature, the application of the original framework in a specific context is to examine whether contextual factors cause the variations in TPB’s prediction [146]. Focusing on green consuming behaviours, YurievDahmen [9] also argued a study significantly contributed to the literature by examining contextual adaptations of the original TPB in diverse settings due to the impact of contextual factors causing the variations. This analysis confirms that, despite potential variances introduced by the unique characteristics of Generation Z consumers living in an emerging and collectivistic country, the original TPB effectively predicts green consumption behaviours. This study contributes to the theoretical development by securing the contextual adaptations of TPB in diverse settings.

Second, one criticism of TPB in predicting green behaviour is that the original models only focus on the direct relationship between key variables and sufficiently ignore how the key variables interact with one another [9, 147]. Although TPB does not inherently assume mediation among its core variables [29], recent empirical results adequately support evidence for the mediating effects of key variables. For instance, Mishra and Kaur [30] confirms the mediating of green attitude on the green subjective norms and green purchasing intentions association. Similarly, VuHa [31] found that green perceived behavioural control mediates the green subjective norms - green purchasing intentions association. Lastly, AlagarsamyMehrolia [32] showed the mediating role of green purchasing intentions on the green attitude - green purchasing behaviour association. This study draws upon the original framework to indicate the mediating role of green purchasing intentions on the link between three key predictors (e.g., green attitude, green perceived behavioural control, green subjective norms) and the dependent variables (e.g., green purchasing behaviours). In this regard, this study significantly extends the current understanding of TPB by emphasising the interaction between key variables as well as the mechanism by which how three key predictors impact behaviours indirectly.

### 5.2. Practical implications

Drawing upon the findings, practical implications for policymakers and firms, which aim to enhance the promotion of green purchasing behaviours among Vietnamese Generation Z consumers, emphasise the integration of collectivistic values, Generation Z characteristics, and economic developments in Vietnam.

First, policymakers are urged to initiate community-based environmental programs which capitalise on collectivistic cultural values [148]. Encouraging Vietnamese Generation Z individuals to participate in such programs in order to foster collective spirit effectively, and therefore, significantly materialising green actions creating positive environmental and social change [148, 149]. Furthermore, policymakers should promote environmental educational campaigns which emphasise the communal benefits of eco-friendly living because it is significantly aligned with Generation Z’s environmental values [69]. Economic incentives such as subsidies for purchases of eco-friendly products or tax benefits for companies practising green production, which facilitate economically accessible environmentally responsible choices, are essential [150]. Moreover, policies relating to financial incentives for the development of green infrastructure, such as subsidies for renewable energy projects or tax breaks for companies, should be encouraged in order to increase the availability of infrastructure supporting green purchasing [151]. Those policies are critical for enabling environmentally friendly consumption among Vietnamese Generation Z individuals.

Firms engaged in executing marketing campaigns should tailor their strategies to emphasise the social and environmental benefits of their products, which resonate deeply with the collectivistic and environmentally aware traits of Generation Z. The strategic use of digital platforms, which involves leveraging social media influencers who advocate for environmental causes and creating engaging content that fosters environmental awareness, is crucial [152]. In addition, firms are encouraged to pursue collaborative marketing efforts with other businesses, which can significantly extend the reach and impact of their green marketing initiatives to promote the norms of green consumption [153]. Ensuring the accessibility of eco-friendly products, which entails enhancing distribution strategies to make these products readily available and convenient for purchase, particularly through online platforms supported by eco-friendly delivery options, is imperative [154]. These marketing strategies should aim not only to promote green products but also to integrate these into the daily lives of consumers, which will help cement eco-conscious purchasing as a normative behaviour among Vietnamese Generation Z consumers.

## 6. Limitations and directions for future studies

This study investigates the factors influencing green purchasing intentions and green purchasing behaviours among Generation Z in Vietnam. While the findings extend current knowledge and offer practical insights, several limitations must be acknowledged, which in turn present opportunities for upcoming research. First, the focus of this study on Vietnamese Generation Z limits its generalizability. The unique contextual factors such as the collectivistic culture, Generation Z values, and the economic progress of Vietnam significantly influence the impact of attitudes, subjective norms, and perceptions of behavioural control on intentions and actual behaviours. Consequently, the results may not generalise to other generations, cultural groups, or regions with different economic developments. Future studies should replicate this model in contexts that share significant similarities with Vietnam to validate the study’s results further. Second, the research employs a cross-sectional approach, which makes it challenging to establish causal relationships between variables. Cross-sectional data only provide a snapshot at a single point in time and cannot capture dynamic relationships or changes in behaviours and intentions over time. This limitation is critical as it impedes our ability to establish causality. Future studies could benefit from using longitudinal or experimental methods, which would allow for examining how changes over time affect these variables and better establish causality. Third, this study aims to shed light on the interrelationships between key variables within the TPB framework in the context of Vietnamese Generation Z consumers due to previously mixed findings. While TPB successfully describes the factors influencing intentions and behaviours in this setting, future studies should consider the following recommendations to further illuminate the drivers of green consumption. Another research avenue for the theoretical development of TPB is to integrate variables related to environmentalism in addition to the key TPB variables. Moreover, there is limited understanding of the antecedents of attitude, subjective norms, and perceived behavioural controls within the TPB framework. Integrating other theories, such as attribution theory, could address these antecedents. By doing so, future studies could significantly illuminate the complex relationships and mechanisms driving intentions and actual behaviours related to green consumption.

## Supporting information

S1 File Dataset. (CSV)

S2 File Research instruments. (DOCX)
