# Supplementary material for: Exploring green purchasing intentions and behaviours among Vietnamese Generation Z: A perspective from the theory of planned behaviour
Source: PLoS One. 2025 May 28;20(5):e0323879. doi: 10.1371/journal.pone.0323879 (PMC12118922; doi:10.1371/journal.pone.0323879)
Supplement: S2 File — (DOCX) [file pone.0323879.s002.docx]

**Main questions**

**Green attitude**

I prefer green products because they satisfy my values
I prefer green products because it is environment friendly

I believe that the green products are competitive
It is exciting for me to buy green products

**Green subjective norm**

Most people who are important to me think that I should purchase environmentally friendly products.

Most people who are important to me would approve of me purchasing environmentally friendly products.

My household/family members think I ought to be purchasing environmentally friendly products.

My friends/colleagues think I ought to be purchasing environmentally friendly products.

**Green perceived behavioral control**

I believe I have the ability to purchase green products.

If it were entirely up to me, I am confident that I will purchase green products.

I see myself as capable of purchasing green products in future.

I have resources, time and willingness to purchase green products

**Green purchasing intention**

I am keen to purchase green products.

I am keen to search for green products.

I am keen to choose green products over non- green products.

I am keen to switch to green products from non- green products

**Green purchasing behavior**

When shopping, I deliberately check products for environmentally harmful ingredients.

When shopping, I deliberately choose products with environmentally friendly packaging.

I will choose to buy environment-friendly products, even if they are more expensive than other products.

When shopping, when I consider buying a product, I will look for a certified environmental label.

**Demographic question**

1. Please indicate your gender:

- Male
- Female

1. Please indicate your age: ......................................................................
2. Please indicate your highest level of education

- Secondary school
- High school
- Undergraduate
- Graduate

1. Please indicate your marital status

- Single
- Married
